# Parathyroid Function Saving Total Thyroidectomy Using Autofluorescence and Quantified Indocyanine Green Angiography

**DOI:** 10.1089/ve.2021.0008

**Published:** 2021-06-10

**Authors:** Milou E. Noltes, Madelon J.H. Metman, Liesbeth Jansen, Emma W.M. Peeperkorn, Anton F. Engelsman, Schelto Kruijff

**Affiliations:** Department of Surgery, University of Groningen, University Medical Center Groningen, Groningen, The Netherlands.; Department of Nuclear Medicine and Molecular Imaging, University of Groningen, University Medical Center Groningen, Groningen, The Netherlands.; Department of Surgery, Amsterdam UMC, University of Amsterdam, Amsterdam, The Netherlands.

**Keywords:** parathyroid, ICG angiography, hypoparathyroidism, autofluorescence, total thyroidectomy

## Abstract

***Introduction:*** Postoperative hypoparathyroidism is one of the most common complications after total thyroidectomy. In recent years, several techniques have been employed, trying to save parathyroid glands during thyroid surgery, such as autofluorescence and indocyanine green (ICG) angiography. In this study, we present a systematic approach to a parathyroid function saving total thyroidectomy using autofluorescence and quantified ICG angiography.

***Materials and Methods:*** Step-by-step video demonstration of a total thyroidectomy for thyroid cancer utilizing parathyroid autofluorescence and ICG angiography.

***Results:*** A systematic step-wise approach to a total thyroidectomy using autofluorescence and quantified ICG angiography is demonstrated. The set moments of deployment, settings of the camera, and a standardized workflow model for parathyroid autofluorescence and ICG angiography are noted.

***Conclusion:*** A systematic approach to parathyroid autofluorescence and quantified ICG angiography during total thyroidectomy may eventually guide the surgeon in early identification of the parathyroid glands and the need for parathyroid autotransplantation, thereby predicting and preventing postoperative hypoparathyroidism.

The authors have no related personal conflicts of interest to declare that could be perceived as prejudicing the impartiality of the research reported. For this study, the Quest Spectrum was used. The authors have no conflicts with this or any other commercial entity. This research did not receive any specific grant from any funding agency in the public or commercial sector.

Runtime of video: 9 mins 59 secs

This video was presented at the Third Symposium on Parathyroid Fluorescence 2021.

## Graphical abstract

**Figure:**
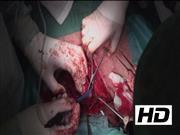

**Figure:**
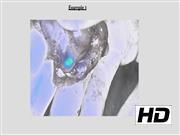

**Figure:**
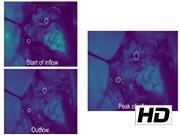

## Video

## Files

**MP4:** 

**PDF:** 

